# A Peculiar Case of Invasive *Streptococcus pneumoniae*

**DOI:** 10.1155/2017/1530507

**Published:** 2017-12-31

**Authors:** Adeel Rafi Ahmed, Liam Townsend, Helen Tuite, Catherine Fleming

**Affiliations:** Department of Infectious Disease, University Hospital Galway, UCHG, Newcastle Rd., Galway H91YR1, Ireland

## Abstract

Patients commonly present to the emergency department with acute respiratory distress; however, the differentials are broad and at times difficult to distinguish. We describe a case of severe community-acquired pneumonia (CAP) secondary to invasive *Streptococcus pneumoniae*. The patient was intubated within 3 h of presentation and suffered multiorgan failure within 72 h of intensive care unit (ICU) admission. This case is a stark illustration of how the most common bacteria associated with CAP can be fatal and highlights the associated markers of severity. It also outlines other potential complications including a very rare phenomenon of cardiomyopathy with myocarditis associated with *S*. *pneumoniae* bacteraemia.

## 1. Case Presentation

A 52-year-old female presented to the emergency department (ED) resuscitation unit with a 5-day history of progressive shortness of breath and productive cough of green sputum. She described some brief episodes of hot and cold spells but had no documented fever or rigors. She was too tachypnoeic to further offer any history. Vitals on presentation were as follows: pulse oximeter reading of 78% on room air, heart rate (HR) of 110 bpm, blood pressure of 85/60 mmHg, respiratory rate of 37 breaths per minute, and temperature of 35.4°C. Initial management was commenced by the ED physicians.

A brief collateral history was obtained from her daughter. The patient was visiting Ireland on holiday and had arrived 6 days ago from Minnesota, USA. Her past medical history included chronic migraine, genital herpes, and zika virus infection, which was acquired 2 months ago during a visit to Mexico and was treated supportively. She was an ex-smoker with 10-year pack history, and her alcohol intake was described as moderate by her daughter. Her medications included hydrocodone, topiramate, and gabapentin all of which she had been on for more than 2 years. She had no history of staying in motels, cruise ships, exposure to birds, purchase of new pets, sick contact, intravenous drug usage, or tick bites.

**Figure 1:**
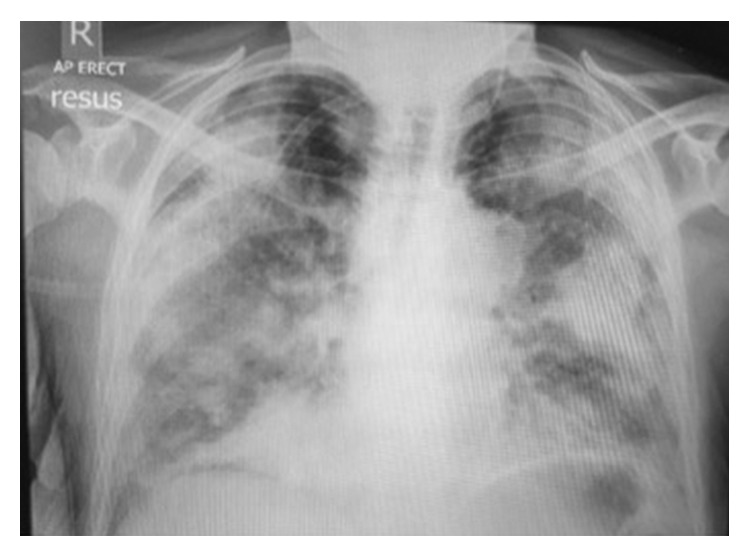
Chest radiograph showing multiple foci of consolidation representing multifocal pneumonia or possible pulmonary infarcts secondary to multiple pulmonary emboli.

Her chest radiograph (Figure 1) showed multiple foci of consolidation representing multifocal pneumonia or possible pulmonary infarcts secondary to multiple pulmonary emboli.

Blood results (Table 1) showed a CRP of 516 mg/L, urea of 28 mmol/L, creatinine of 163 *μ*mol/L, and D-dimer of 1400 ng/ml and a mixed respiratory metabolic acidosis with type 2 respiratory failure on arterial blood gas. Microbiology on call was contacted, and she was commenced on broad spectrum coverage with piperacillin-tazobactam, clarithromycin, vancomycin, and oseltamivir. Bilevel positive airway pressure (BIPAP) as a form of noninvasive ventilation was commenced, with ICU involvement.

**Figure 2:**
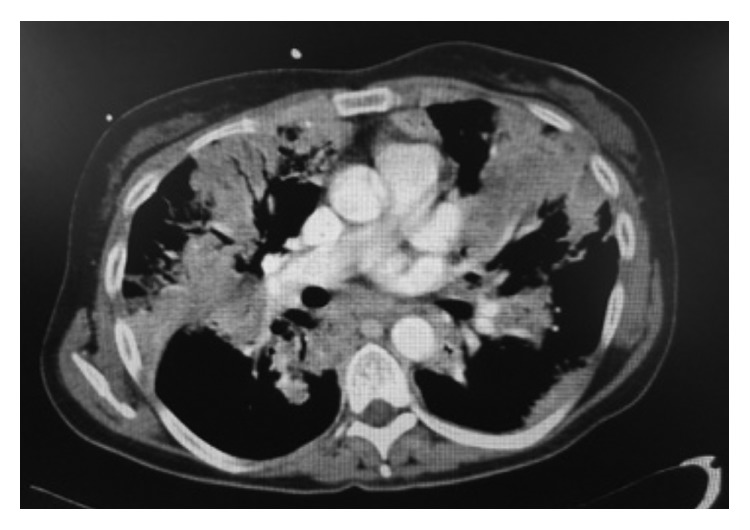
CT-pulmonary angiogram showing extensive multifocal consolidations, reactive mediastinal lymphadenopathy with no pulmonary embolism.

An urgent CT-pulmonary angiogram was arranged (Figure 2) and showed no acute pulmonary emboli, extensive multifocal consolidations, and most likely reactive mediastinal lymphadenopathy.

Her arterial blood gases continued to deteriorate, and 3 h after admission she was severely acidotic. She was intubated and moved to the ICU. Her initial ICU management was based around a working diagnosis of acute respiratory distress syndrome secondary to severe CAP.

The Infectious Disease (ID) team was consulted the following morning, and her antibiotics were further rationalised to ceftriaxone to cover invasive *S. pneumoniae*; vancomycin to cover resistant *S*. *pneumoniae* and community acquired MRSA; ciprofloxacin to cover atypical pneumonia including legionellosis; and oseltamivir for influenza. Twenty-four hours after her admission, blood cultures result came back positive for *S*. *pneumoniae*, and she was deescalated to high-dose ceftriaxone therapy.

Forty-eight hours after admission, she developed severe pulmonary oedema, and her troponin T reached a peak of 20,643 ng/L and proBNP of 68,543 pmol/L. An urgent angiogram demonstrated no coronary artery disease. Echo showed posteroapical akinesia with an ejection fraction of 40–45% with no valvular abnormality. The working diagnosis of cardiomyopathy with myocarditis secondary to *S*. *pneumoniae* was established by the cardiology and ID team.

Seventy-two hours after admission, she was anuric with worsening renal function despite optimal fluid management. She was commenced on continuous venovenous haemofiltration (CVVH). Despite these complications and the development of multiorgan failure, she responded to the antibiotic regime and was successfully extubated with no further requirement for invasive ventilation or CVVH. She developed extensive critical illness myopathy and is currently undergoing rehabilitation.

## 2. Discussion

This case is a stark illustration of how *S*. *pneumoniae*, the most common bacteria associated with CAP, can be potentially fatal. The presentation of the patient was consistent with severe pneumonia; however, the initial radiological reports pointed to a broader differential including infection, infarction (pulmonary embolism), and inflammation (vasculitis with pulmonary haemorrhage).

The patient's travel history provided another dilemma for the on-call admitting physician. In USA, community-acquired MRSA is common, while in Ireland this would be considered very rare. Exposure to other travellers and hotels meant viral and atypical bacterial aetiology had to be kept in mind when deciding the empiric treatment regime. Once the blood cultures grew *S*. *pneumoniae*, antibiotics were further rationalised. It is important to note that the patient was HIV, hepatitis B, and hepatitis C negative.

Invasive pneumococcal disease is defined as an infection confirmed by the isolation of *S*. *pneumoniae* from a normally sterile site (e.g., blood or cerebrospinal fluid but not sputum) [1].

Invasive pneumococcal disease carries a mortality rate of 15–20%, the majority of which are within the first 72 hours, regardless of primary cause. Once a patient is admitted to ICU secondary to pneumonia (of any causative agent), the mortality rate goes up to 50% [2]. It is important to note that the patient demonstrated many of the poor prognostic factors associated with CAP. This includes hypoxemia (PaO_2_ < 8 kPa), urea > 7 mmol/L, respiratory rate > 30 breaths per minute, blood pressure systolic < 90 mmHg, and perhaps diagnostically most relevant, a positive blood culture which is seen in 10% of pneumonia cases of which approximately 60% are *S. pneumoniae* [2]. The patient was also in type 2 respiratory failure, a marker for a tiring patient and which should prompt the early involvement of ICU and possible intubation [2].

*S*. *pneumoniae* infection is associated with alcoholism [3], cigarette smoking, diabetes, chronic organ failure (e.g., chronic obstructive pulmonary disease (COPD)/heart failure), influenza infection, hyposplenism, and age less than 2 or more than 65. In our case, the patient had a history of smoking and moderate alcohol intake.

In cases of invasive *S*. *pneumoniae*, secondary complications such as arthritis, meningitis, endocarditis, and very rarely severe transient cardiomyopathy with myocarditis, as demonstrated in this case, may occur; the primary physician should have a high diagnostic suspicion for them [3, 4].

## Figures and Tables

**Table 1:** Pertinent laboratory findings.

Parameters	Normal range	Presentation	3 h after presentation	Day 3
pH	7.35–7.45	7.23	6.96	—
Lactate	0.5–1.5 mmol/L	2.6	1.8	—
PaO_2_	>10.6 kPa	5.7	7.9	—
PaCO_2_	4.7–6 kPa	6.5	12.1	—
HCO_3_	22–28 mmol/L	20.3	20.5	—
Sodium	135–145 mmol/L	137	—	140
Potassium	3.5–5.0 mmol/L	4.2	—	5.9
Urea	2.5–6.7 mmol/L	28	—	31
Creatinine	70–50 *μ*mol/L	163	—	260
CRP	<10 mg/L	**516**	—	140
Troponin T	0–14 ng/L	8	—	**20,643**
ProBNP	0–500 pmol/L	—	—	**68,354**
White cell count (total)	4–11 × 10^9^/L	11.3 × 10^9^	—	11 × 10^9^
Haemoglobin (Hb)	Men: 13.0–18.0 g/dL	14.7	—	12
	Women: 11.5–16.0 g/dL			
Platelets	150–400 × 10^9^/L	190 × 10^9^	—	300 × 10^9^
D-dimer	<400 ng/ml	1400	—	—
INR	1–1.2	1.0	—	—

## Additional Points

1. Blood cultures should be carried out in all patients suspected of pneumonia as a positive blood culture is a marker of severity and helps guide antimicrobial therapy especially in severe cases.
2. *Streptococcus pneumoniae* is the commonest pathogen associated with CAP and however carries a high mortality especially once bacteraemia (invasive *S*. *pneumoniae*) is present.
3. A high suspicion of index for secondary complication such as endocarditis/meningitis and rarely cardiomyopathy with myocarditis needs to be considered once bacteraemia is present.
